# Exploring the Gender Difference and Predictors of Perceived Stress among Students Enrolled in Different Medical Programs: A Cross-Sectional Study

**DOI:** 10.3390/ijerph17186647

**Published:** 2020-09-11

**Authors:** Carmenrita Infortuna, Francesco Gratteri, Andrew Benotakeia, Sapan Patel, Alex Fleischman, Maria Rosaria Anna Muscatello, Antonio Bruno, Rocco Antonio Zoccali, Eileen Chusid, Zhiyong Han, Fortunato Battaglia

**Affiliations:** 1 Department of Biomedical and Dental Sciences and Morphofunctional Imaging, Policlinico Universitario, University of Messina, 98124 Messina, Italy; carmen.infortuna@gmail.com (C.I.); francescogratteri92@icloud.com (F.G.); mmuscatello@unime.it (M.R.A.M.); antonio.bruno@unime.it (A.B.); zoccali@unime.it (R.A.Z.); 2 Department of Medical Sciences and Neurology, Hackensack Meridian School of Medicine at Seton Hall University, Nutley, NJ 07110, USA; andrew.benotakeia@student.shu.edu (A.B.); Zhiyong.han@hackensackmeridian.org (Z.H.); 3 Department of Pre-Clinical Sciences, New York College of Podiatric Medicine, New York, NY 10035, USA; sapanppat@gmail.com (S.P.); afleischman2023@nycpm.edu (A.F.); echusid@nycpm.edu (E.C.)

**Keywords:** stress, medical student, temperament, self-esteem, optimism

## Abstract

Female medical students seem to experience higher level of perceived stress. Moreover, there is a lack of research examining perceived stress in students enrolled in different medical programs. We analyzed the association between temperament traits, optimism, self-esteem, and perceived stress of students pursuing a Doctor of Medicine (MD) degree and students pursuing a Doctor of Podiatric Medicine (DPM) degree. A cross-sectional study was conducted of two cohorts: allopathic medical students (N = 154) and the podiatric medical students (N = 150). Students anonymously completed the Perceived Stress Scale (PSS-10), Temperament Evaluation of Memphis, Pisa, Paris, and San Diego Auto Questionnaire (TEMPS-A), Rosenberg Self-Esteem Scale, and Life Orientation Test—Revised (LOT-R). We analyzed differences in the two cohort of students and predictors of perceived stress. There were no differences in the overall perception of stress between both cohorts (allopathic medical students: 18.83 ± 0.56; podiatric medical students: 19.3 ± 0.72; *p* = 0.4419). Women reported higher perceived stress in both programs (allopathic medical students: *p* = 0.0.038; podiatric medical students: *p* = 0.0.038). In both allopathic and podiatric medical students, the cyclothymic temperaments and anxious traits were positive predictors while hyperthymic temperaments and optimism traits were negative predictors of perceived stress. The level of perceived stress experienced by students pursuing different doctoral degrees in healthcare is similar. Regardless of the curriculum differences, female students experience higher perceived stress and there is evidence for similarities in predictors amongst allopathic and podiatric medical students.

## 1. Introduction

In the first two years of graduate medical education, students enrolled in medical schools offering Medical Doctors (MD) degrees and Doctor of Podiatric Medicine (DPM) degrees are introduced to a well-rounded curriculum that encompasses foundational subjects—biochemistry, histology, and immunology—and basic clinical knowledge—pharmacology, radiology, and neuroscience—for preparation for introductory board examinations. Upon a successful completion of board examinations, students continue through a rigorous clinical curriculum spanning two additional years followed by subsequent board examination [1,2]. However, podiatric medical school students hone their specialty with specialized surgical and podiatric medicine courses and clinical hours prior to post-graduate residency [3]. Unlike American medical education, European medical education is a six-year education program. Both American and European medical education require post-medical education residency [4].

Medical students experience a high degree of psychological stress due to frequent examinations, vastness of curriculum, length of training, and high tuition costs [5]. This cumulative effects of stressful experiences in daily life (allostatic loads) may interfere with one’s goal [6] and may lead to the development of anxiety and depression over time [7]. Previous studies indicate that female medical students [8,9,10] experience higher level of distress. Furthermore, the data in the literature are not always consistent [11,12,13,14] and the association of gender with the perception of stress in medical student is still be addressed. Thus far, studies comparing the perception of stress in medical students enrolled in different medical programs are lacking. Furthermore, it is yet to be investigated whether the predictors of perceived stress are consistent within the different medical student population.

Stress theory examines the relationship between subjective stress and its contributing factors. In the Transactional Stress model, subjective stress results when threatening and environmental demands of a stressor exceed one’s coping resources [15]. Varying perceptions of stress differ from person-to-person even when exposed to the same objective stressor. Thus, several additional factors, such as temperament, optimism, and self-esteem could be associated with the perception of stress and the ability to develop coping strategies. Using the Temperament Evaluation of Memphis, Pisa, Paris, and San Diego-auto-questionnaire short version (TEMPS-A), five temperament dimensions can be described: cyclothymic, depressive, irritable, hyperthymic, and anxious [16]. Previous studies have reported the association between mental distress and specific emotional temperaments and character traits [17,18,19,20,21,22]. Furthermore, optimism is one’s psychological view of life and its possible outcomes. An optimistic perspective drives a friendly, pleasant demeanor and a positive outlook on goals, to which a pessimistic perspective antagonizes [23,24]. Another important factor is self-esteem, that refers to one’s view of self-worth. A positive self-esteem increases one’s attitude towards themselves, others, and future goals, to which negative self-esteem antagonizes [25]. Low self-esteem increased negative and harmful emotions towards oneself and is associated with stress [26].

To date, there is no study comparing specific mental health factors of graduate medical students enrolled in allopathic and podiatric medical schools. The objectives of this study aimed to compare the following in a cohort of allopathic and podiatric medical school: (i) gender differences in perceived stress levels; (ii) temperament states (cyclothymic, depressive, irritable, hyperthymic, and anxious); (iv) optimism; and (v) self-esteem. The study compared students enrolled in different medical programs (different length of time to graduation, admission requirements, language of study, nationality, medical degree titles, and the influence of culture and tradition) to determine if perceived stress is intrinsic to the medical profession and its education; therefore remaining unaffected by differences in culture or curriculum.

## 2. Methods

A Cross-sectional study was conducted of two cohorts of students at the allopathic School University of Messina, in Messina, Italy, and the New York College of Podiatric Medicine in New York City, New York. The study was approved by the Institutional Review Boards of the University of Messina and The New York College of Podiatric Medicine and was conducted between June and September 2019. The target population included all students attending preclinical years (the first and second year) at the students of University of Messina Medical School (N = 586) and at the New York College of Podiatric Medicine (N = 190). The study was announced through flyers and social media. Participants were issued an anonymous questionnaire that was returned in a sealed envelope and deposited in a box outside the classroom. The survey contained a statement of consent and the participation was voluntary and anonymous. Population frequencies and variables examined included age, gender, grade point average (GPA), temperament traits, self-esteem, optimism, and perceived stress.

### 2.1. Measures

Affective temperament traits were evaluated by the Temperament Evaluation of Memphis, Pisa, Paris, and San Diego Auto Questionnaire (TEMPS-A) short version [16]. TEMPS-A questionnaire short version is a 39-question instrument to which participants answer “yes” (1) or “no” (2) [16]. The evaluator scores five subscales (cyclothymic, dysthymic, irritable, hyperthymic, and anxious). The TEMPS-A has good psychometric characteristics [27].

The Rosenberg Self-Esteem Scale quantitatively measures the self-worth of the participant. The Rosenberg Self-Esteem Scale is a 10-question survey which is scored from the extent to which the participants agreed with the questions (from “strongly agree” to “strongly disagree”) [28]. Self-esteem correlates to a high final value [28]. The instrument has good psychometric properties [29].

The Life Orientation Test-Revised (LOT-R) quantitatively measures the optimism of the participant. The LOT-R is a 10-questions survey to which four items are not counted. Questions are given a rating from “I agree a lot” to “I disagree a lot” [30]. Psychometric properties were found to be satisfactory [31].

The Perceived Stress Scale—10 (PSS-10) relays quantitative levels of perceived stress [32]. The PSS-10 is a 10-item instrument to which participants rate each listed question about feelings during the past month on a Likert scale (0–4). The PSS-10 has high validity and reliability in medical student population [33].

### 2.2. Statistical Analysis

Descriptive statistics were conducted to outline medical student’s demographic information. Independent *t*-tests were performed to analyze difference in temperament traits scores, optimism, and self-esteem between allopathic medical students and podiatric medical students. Furthermore, predictors of perceived stress were identified by using two multiple regression models (Model 1: allopathic medical students; Model 2: podiatric medical students). Alpha was set at 0.05 for all statistical tests. Statistical analysis was performed using the Statistical Package for Social Sciences (SPSS, IBM, Armonk, NY, USA) for Windows (version 24.0).

## 3. Results

The sample consisted of 304 subjects (154 allopathic medical students and 150 podiatric medical students). For allopathic medical students, the mean age of students was 20.88 ± 1.43 with 53.3% of students being female and 46.7% being male. Conversely, podiatric medical students were slightly older (m = 24.17 ± 3.03) with similar gender proportions (60% Female, 40% Male). Lastly, the most significant difference in demographics was ethnicity with allopathic medical students being 100% Caucasian and podiatric medical students being 54% Caucasian, 34% Asian, 10% Hispanic, and 2% African American.

Eight unpaired *t*-Tests were conducted to compare temperament traits scores, self-esteem, optimism, and perceived stress between both cohorts (Table 1). There was no difference in PSS-10 score between the two cohorts (allopathic medical students: 18.83 ± 0.56; podiatric medical students: 19.3 ± 0.72; *p* = 0.4419). In both group of students, women displayed higher level of perceived stress (allopathic medical students: *t* = 1.8′; *p* = 0.041; podiatric medical students: *t* = 2.23; *p* = 0.0.038). Both allopathic and podiatric medical students showed no significant difference in temperament trait scores: hyperthymic (*p* = 0.7080), cyclothymic (*p* = 0.8594), depressive (*p* = 0.7417), irritable (*p* = 0.4106), and anxious (*p* = 0.1422). Similarly, it was found that both allopathic and podiatric medical students showed the same levels of optimism (*p* = 0.4601) and perceived stress (*p* = 0.4419). Conversely, podiatric medical students displaced higher self-esteem than allopathic medical students (*p* = 0.0036).

Lastly, linear regressions were conducted for both cohorts to determine predictors for perceived stress in allopathic (Model 1) and podiatric (Model 2) medical students. For Model 1, a significant regression equation was found (F_(11,156)_ = 13.6, *p* < 0.0001) with an R^2^ of 0.51. The individual predictors were examined further and indicated that cyclothymic (*p* < 0.000) and anxious traits (*p* = 0.002) were positive predictors of perceived stress. On the contrary, hyperthymic trait (*p* = 0.001) and Lot-R scores (*p* = 0.002) were negative predictors (Table 2). Similar results were found when analyzing predictors for perceived stress in podiatric medical students. Model 2 was significant (F_(12,149)_ = 10.7, *p* < 0.0001) with an R^2^ of 0.48. Both cyclothymic (*p* = 0.03) and anxious (*p* < 0.0001) temperament scores were positive predictors of perceived. Conversely, both hyperthymic temperament (*p* < 0.0001) and Lot-R scores (*p* = 0.045) were negative predictors of perceived stress for podiatric medical students (Table 3). Self–esteem, depressive, and irritable traits showed no significant predictive value for perceived stress in both models.

## 4. Discussion

Both allopathic and podiatric medical students, despite pursuing different medical programs leading to professions in healthcare with different scopes of practice, showed the same levels of perceived stress. Furthermore, both cohorts of students showed the same predictors of perceived stress. In both cohorts, perceived stress levels were higher in women.

Stress is a determining factor in student well-being and mental health [34]. While the prevalence of stress in allopathic medical students has previously been reported [5,34], investigation of stress in podiatric medical students is lacking. Only one previous study analyzing podiatric medical student well-being revealed a high prevalence of perceived stress, poor sleep quality and excessive daytime sleepiness [14]. Thus, our results fill a gap in the literature and provide, for the first time, comparative data in students pursuing different health professions. Our results confirm previous reports indicating that female medical students show higher level of perceived stress [8,9]. We can only speculate regarding possible determinants. Gender-specific differences in stress reactivity and consequent higher predisposition to depression and anxiety have been previously identified in women [35]. It has been suggested that differences in neuroendocrine and hypothalamic–pituitary–adrenal (HPA) axis reactivity to stress may underlie the higher perception of stress in women [36]. Since both allopathic and podiatric medical students showed similar scores in temperament traits (cyclothymic, depressive, irritable, hyperthymic, and anxious) and similar optimism levels, our results direct towards the importance of stressors rooted in medical school culture rather than difference in burden due to specific curricula (e.g., program duration, scope of practice, type of curriculum, workload, grading system, teaching model, class size, public or private, and administrative resources).

We also found evidence for similar predictors of perceived stress in students enrolled in different programs. For instance, in a clinical population, hyperthymic temperament trait, characterized by having an abnormally positive emotional reaction to daily challenges, has been shown to be a negative predictor for perceived stress [16]. This could possibly be due to fact that individuals with high score in hyperthymic traits are warm, people-seeking, overoptimistic, and exuberant [37]. Thus, those with this trait may be more likely to cope with stressors by sharing problems with family and/or friends, rather than enduring the stressor alone. Additionally, those with higher levels of actionability and responsibility can have a more rapid and effective response to a stress [16]. Optimism has been found to positively influence mental health by counteracting the effect of stress [38] and, in our study, was a negative predictor of perceived stress. Studies by others have indicated that a positive attitude toward life events decreases cortisol secretion and autonomic activation [39,40,41]. Our result is of pivotal importance since a variety of psychological treatments can boost optimism [42,43], and they could be integrated in psychological support program tailored to reduce abnormal medical student’s reaction to the stressful situation.

Cyclothymic temperament trait, characterized by the tendency to quickly change emotional moods, was found to be a positive predictor for increased perceptions of stress. It was shown that cyclothymic temperaments are associated with improper behavioral regulation under naturally occurring stress [17]. The idea that dysregulation of emotions is linked to a greater perception of stress is further supported by the relationship between anxious trait and perceived stress in both groups of students in our study. Anxious temperament trait is characterized by disproportionate levels of nervousness, fear, and apprehension. It was found that those who score high on this trait were unable to discriminate between realistic implications of stressors and exaggerate behavioral responses, and they remain fearful and apathetic towards possible coping strategies [44].

Our results align with proposed theories of stress [15]. This finding also opens an exciting avenue: both hyperthymic temperament and dispositional optimism can predispose students to utilizing more effective coping mechanisms while cyclothymic and anxious traits lead to a self-destructive and harmful coping tactics [45]. Given that our findings show that these effects are largely independent of curricula structure, we believe that they reflect student’s response to stressors inherent to medical culture.

This study has some limitation. We employed a cross-sectional study design and, thus, all findings are associations. To determine causality, a prospective study would need to be conducted. Additionally, a study involving a larger number of subjects should be planned to generalize our findings. We should also note the need of a study investigating the impact of different stressors.

## 5. Conclusions

Our data indicate that, by developing a more optimistic environment in medical schools, allopathic and podiatric medical students would be able to develop a higher resiliency and have less perception of stress. Supporting psychological programs are often utilized for positively impacting psychological and emotional wellbeing of medical students [46]. Because of similarities found between the cohorts in our study, it would be conceivable to design and implement psychological programs incorporating optimism training to be shared by different institutions. Students enrolled in different medical programs could benefit from this experience. In addition, in view of the higher perceived stress level reported in women, future studies are warranted to investigate gender-specific stressors and coping strategies.

## Figures and Tables

**Table 1:** Scores obtained on the five dimensions of the Temperament Evaluation of Memphis, Pisa, Paris, and San Diego questionnaire short version (TEMPS-A), the LOT-R scale, and the Rosenberg Self-Esteem Scale in the allopathic medical students and podiatric medical students. Data are reported as mean ± SE. (**) *p* < 0.01.

Variables	*t*-Value	*p*-Value
PSS-10	−0.770	0.4419
Cyclothymic	0.177	0.8594
Depressive	0.330	0.7417
Irritable	0.824	0.4106
Hyperthymic	−0.375	0.7080
Anxious	−1.471	0.1422
LOT-R	0.740	0.4601
Rosenberg scale	−2.933	0.0036 **

**Table 2:** Multiple regression analysis (Model 1). Predictors of perceived stress in allopathic medical students.

Variables	Unstandardized Coefficients		Standardized Coefficients	*t*	Sig.	95% Confidence Interval	
	B	Std. Error	Beta			Lower Bound	Upper Bound
(Constant)	13.443	10.306		1.304	0.194	−6.929	33.815
Gender	−0.732	0.914	−0.049	−0.801	0.425	−2.538	1.075
Age	0.296	0.318	0.057	0.933	0.353	−0.332	0.925
Relatives	−0.802	1.063	−0.048	−0.755	0.452	−2.903	1.299
GPA	0.247	0.26	0.057	0.95	0.344	−0.268	0.762
Cyclothymic	0.507	0.101	0.44	5.04	0.000	0.308	0.706
Depressive	0.437	0.219	0.223	1.993	0.058	0.004	0.871
Irritable	−0.47	0.282	−0.259	−1.665	0.098	−1.029	0.088
Hyperthymic	−0.621	0.188	−0.374	−3.294	0.001	−0.993	−0.248
Anxious	1.06	0.339	0.232	3.131	0.002	0.391	1.729
Lot-R	−0.305	0.095	−0.199	−3.221	0.002	−0.492	−0.118
Rosenberg scale	−0.126	0.097	−0.089	−1.292	0.199	−0.318	0.067

Dependent Variables: PSS-10, N = 154.

**Table 3:** Multiple regression analysis (Model 2). Predictors of perceived stress in podiatric medical students.

	Unstandardized Coefficients		Standardized Coefficients	*t*	Sig.	95% Confidence Interval for B	
Ind. Variables	B	Std. Error	Beta			Lower Bound	Upper Bound
(Constant)	7.342	5.411		1.357	0.177	−3.359	18.042
Gender	−0.184	0.719	−0.016	−0.256	0.799	−1.606	1.238
Age	−0.08	0.13	−0.039	−0.614	0.54	−0.337	0.177
Ethnicity	−0.24	0.328	−0.047	−0.731	0.466	−0.889	0.409
Relatives	0.863	0.931	0.058	0.927	0.356	−0.978	2.704
GPA	0.24	0.835	0.018	0.287	0.774	−1.411	1.89
Cyclothymic	0.352	0.16	0.154	2.198	0.03	0.035	0.669
Depressive	0.066	0.132	0.032	0.497	0.62	−0.196	0.328
Irritable	−0.047	0.144	−0.021	−0.328	0.743	−0.333	0.238
Hyperthymic	1.445	0.248	0.403	5.829	0.000	0.955	1.936
Anxious	1.379	0.255	0.379	5.409	0.000	0.875	1.883
Lot-R	−0.15	0.078	−0.131	−1.94	0.045	−0.304	0.003
Rosenberg scale	0.112	0.073	0.101	1.524	0.13	−0.033	0.257

Dependent Variables: PSS-10; N = 150.
